# Supplementary material for: Mapping hematologists’ HIV testing behavior among lymphoma patients–A mixed-methods study
Source: PLoS One. 2023 Jan 3;18(1):e0279958. doi: 10.1371/journal.pone.0279958 (PMC9810165; doi:10.1371/journal.pone.0279958)
Supplement: S1 Table — (DOCX) [file pone.0279958.s001.docx]

S1 Table. Content of online questionnaire on barriers and facilitators for HIV testing among patients with malignant lymphoma

|  | **Question** | **Response options** |
| --- | --- | --- |
| **Section 1: Respondent characteristics** | | |
| 1.1 | Sex of respondent | Male/Female |
| 1.2 | Age of respondent (in years) | 2-digit field |
| 1.3 | Specialty department of respondent | Pulmonology/gastroenterology/  gynecology/neurology/hematology/  other, i.e.… |
| 1.4 | Job description of respondent | Resident/intern/attending/other, namely… |
| 1.5 | Number of years work experience in this specialty | 2-digit field |
| 1.6 | Type of hospital of respondent | University hospital/teaching hospital/non-teaching hospital/other, i.e.… |
| **Section 2: HIV testing behavior** | | |
| 2.1 | HIV is common in patients in patients with malignant lymphoma | 5-point Likert scale:  Definitely not - Definitely yes |
| 2.2 | I tested patients with malignant lymphoma for HIV in the last year | 5-point Likert scale:  Never - Very often |
| 2.3 | When I see patients with malignant lymphoma, I plan to test them for HIV | 5-point Likert scale:  Strongly disagree - Strongly agree |
| 2.4 | It is likely that I will test patients with malignant lymphoma for HIV in the future | 5-point Likert scale:  Very unlikely – Very likely |
| 2.5 | It's important that I test patients with malignant lymphoma for HIV | 5-point Likert scale:  Very unimportant - Very important |
| 2.6 | It's positive that I test patients with malignant lymphoma for HIV | 5-point Likert scale:  Very negative - Very positive |
| 2.7 | I’m comfortable offering patients with malignant lymphoma an HIV test | 5-point Likert scale:  Very uncomfortable - Very comfortable |
| 2.8 | I'm capable of testing patients with malignant lymphoma for HIV | 5-point Likert scale:  Highly incapable - Highly capable |
| 2.9 | It’s easy testing patients with malignant lymphoma for HIV | 5-point Likert scale:  Very hard - Very easy |
| 2.10 | My colleagues find it important that we test patients with malignant lymphoma for HIV | 5-point Likert scale:  Very unimportant - Very important |
| 2.11 | Patients with malignant lymphoma expect me to test them for HIV | 5-point Likert scale:  Definitely not - Definitely yes |
| 2.12 | HIV testing in patients with malignant lymphoma is discussed in my specialty | 5-point Likert scale:  Never - Very often |
| 2.13 | HIV testing in patients with malignant lymphoma is in our guidelines | 5-point Likert scale:  Definitely not - Definitely yes |
| 2.14 | Ordering an HIV test in patients with malignant lymphoma is easily arranged | 5-point Likert scale:  Very difficult to arrange - very easily arranged |
| 2.15 | HIV testing in patients with malignant lymphoma leads to better health outcomes | 5-point Likert scale:  Definitely not - Definitely yes |
