# Supplementary material for: Mapping hematologists’ HIV testing behavior among lymphoma patients–A mixed-methods study
Source: PLoS One. 2023 Jan 3;18(1):e0279958. doi: 10.1371/journal.pone.0279958 (PMC9810165; doi:10.1371/journal.pone.0279958)
Supplement: S2 Table — (DOCX) [file pone.0279958.s002.docx]

S2 Table. Interview guide for semi-structured interviews with hematologists working in the region of Amsterdam on factors influencing HIV testing behavior among malignant lymphoma patients

| **Part 1: Knowledge on HIV testing recommendations among malignant lymphoma patients** |
| --- |
| What do you know about HIV testing guidelines/recommendations in malignant lymphoma patients? |
| What source(s) have you used to get the information? |
| **Part 2: Attitudes and behavior regarding** **HIV testing among malignant lymphoma patients** |
| What is your attitude/strategy regarding HIV testing in malignant lymphoma patients? |
| Why do you adopt this approach? |
| Do you test for HIV among malignant lymphoma patients presenting with all types of lymphoma? |
| **Part 3: Norms** **regarding** **HIV testing among malignant lymphoma patients** |
| How do your colleagues’ attitudes regarding HIV testing in malignant lymphoma patients influence your approach? |
| How do malignant lymphoma patients’ beliefs on HIV testing influence your attitudes? |
| **Part 4: Self-efficacy regarding** **HIV testing among malignant lymphoma patients** |
| How would you judge your ability to successfully perform HIV testing in malignant lymphoma patients? |
| How would you judge your ability to successfully deliver a positive HIV test result to malignant lymphoma patients? |
| **Part 5: Perceived barriers regarding** **HIV testing among malignant lymphoma patients** |
| What are your perceived barriers for HIV testing in malignant lymphoma patients? |
