# Supplementary material for: Mapping hematologists’ HIV testing behavior among lymphoma patients–A mixed-methods study
Source: PLoS One. 2023 Jan 3;18(1):e0279958. doi: 10.1371/journal.pone.0279958 (PMC9810165; doi:10.1371/journal.pone.0279958)
Supplement: S3 Table — (DOCX) [file pone.0279958.s003.docx]

S3 Table. Interview guide for semi-structured interviews with authors of hematology guidelines working in the region of Amsterdam on the extent of and reasons for HIV testing recommendations in malignant lymphoma guidelines

| **Part 1: Characteristics of HIV testing recommendations in malignant lymphoma guidelines** |
| --- |
| Why is HIV testing included in the malignant lymphoma guidelines? |
| To what extent is HIV testing included in the malignant lymphoma guidelines? |
| What is the evidence for this HIV testing recommendation in the malignant lymphoma guidelines? |
| What are the main characteristics of HIV testing recommendations in malignant lymphoma patients, in terms of clarity, specificity, strength of evidence? |
| What is the process of making the malignant lymphoma guidelines (who made the guidelines, how were they approved and by whom) |
| **Part 2: Communication and dissemination of malignant lymphoma guidelines** |
| What kind of communication strategies did you use for the dissemination and implementation of these malignant lymphoma guidelines? |
| **Part 3: Social system involved in malignant lymphoma guidelines** |
| How did you identify malignant lymphoma guideline users? |
| What kind of targeted user strategies such as feedback did you use to assess the implementation of these malignant lymphoma guidelines? |
