# Supplementary material for: Mapping hematologists’ HIV testing behavior among lymphoma patients–A mixed-methods study
Source: PLoS One. 2023 Jan 3;18(1):e0279958. doi: 10.1371/journal.pone.0279958 (PMC9810165; doi:10.1371/journal.pone.0279958)
Supplement: Attachment — Submitted filename: PONE-D-22-26866 Response to Reviewers.docx [file pone.0279958.s004.docx]

Dr. Simon White

Academic Editor, PLOS ONE

Keele University, UK

Amsterdam, 7 December 2022

Manuscript ref. no.:	PONE-D-22-26866

Title:	Mapping hematologists’ HIV testing behavior among lymphoma patients – A mixed-methods study.

Dear Dr. White,

We thank you kindly for reviewing our manuscript, and for the invitation to revise our work in light of the reviewers’ comments provided.

The comments of the Editor and the Reviewers have been most helpful in improving the manuscript and we thank the reviewers for their time and effort. We hereby submit the revised clean version and a version with tracked changes following their comments.

Please find below our response to the Journal’s requirements and the Editor’s and Reviewers’ original comments in black and italic and our detailed responses to each comment, including any changes, in blue and bold. Where we refer to a manuscript line number, this is to the line number in the version of the manuscript with track changes.

Following your request, we have updated our Competing Interest statement, and the updated statement including confirmation that not alter our adherence to PLOS ONE policies on sharing data and materials can be found below in the point-by-point rebuttal, as well as in the Editorial Manager system under Comments for the Journal Office.

We very much appreciate your consideration of the revised manuscript and hope that it is now suitable for publication in PLOS ONE.

Yours sincerely, on behalf of all co-authors,

Saskia Bogers, MD

Amsterdam University Medical Centers

Department of Infectious Diseases

Meibergdreef 9, 1105 AZ Amsterdam

Phone: +3120 566 5992

Email: s.j.bogers@amsterdamumc.nl

**Journal Requirements:**

1. *Please ensure that your manuscript meets PLOS ONE's style requirements, including those for file naming.*

**Reply: We have reviewed the style requirement files and assured that our files meet the required formatting.**

2. *Please include your ethics statement in the Methods section of your manuscript. In the Methods section of your revised manuscript, please include the full name of the institutional review board or ethics committee that granted ethical approval or exempt the study from ethical approval, the approval or permit number that was issued, and the date that approval/exemption was granted.*

**Reply: This statement was now added at the end of the methods section.**

3. Please provide additional details regarding participant consent. In the ethics statement in the Methods and online submission information, please ensure that you have specified what type you obtained (for instance, written or verbal, and if verbal, how it was documented and witnessed). If your study included minors, state whether you obtained consent from parents or guardians. If the need for consent was waived by the ethics committee, please include this information.

**Reply: These details were now added to the ethics statement at the end of the methods section, and were updated in the editorial manager system.**

4. *Thank you for stating the following in the Competing Interests section:*

*"Dr. Bogers has nothing to disclose. Dr. Zimmermann has nothing to disclose. Dr. Ndong has nothing to disclose. Dr. Davidovich has nothing to disclose. Dr. Kersten reports consulting fees: Kite/Gilead; BMS/Celgene; Novartis; Miltenyi Biotec; Adicet Bio: to institution and payment for honoraria: Kite/Gilead; Roche; BMS/Celgene: to institution and participation on a Data Safety Monitoring Board or Advisory Board: SUBITO study (high dose chemotherapy in breast cancer): No financial compensation. Dr. Reiss reports grants or contracts: Gilead Sciences; ViiV Healthcare; Merck: Investigator-initiated study grants to institution; not related to current work and Participation on a Data Safety Monitoring Board or Advisory Board: Gilead Sciences; ViiV Healthcare; Merck: Honoraria for scientific advisory board participation paid to institution. Dr. Schim van der Loeff has nothing to disclose. Dr. Geerlings has nothing to disclose."*

*Please confirm that this does not alter your adherence to all PLOS ONE policies on sharing data and materials, by including the following statement: "This does not alter our adherence to  PLOS ONE policies on sharing data and materials.” (as detailed online in our guide for authors <http://journals.plos.org/plosone/s/competing-interests>).  If there are restrictions on sharing of data and/or materials, please state these. Please note that we cannot proceed with consideration of your article until this information has been declared.*

*Please include your updated Competing Interests statement in your cover letter; we will change the online submission form on your behalf.*

**Reply: Our updated Competing Interests statement is written below (amended as requested):**

***Dr. Bogers has nothing to disclose. Dr. Zimmermann has nothing to disclose. Dr. Ndong has nothing to disclose. Dr. Davidovich has nothing to disclose. Dr. Kersten reports consulting fees: Kite/Gilead; BMS/Celgene; Novartis; Miltenyi Biotec; Adicet Bio: to institution and payment for honoraria: Kite/Gilead; Roche; BMS/Celgene: to institution and participation on a Data Safety Monitoring Board or Advisory Board: SUBITO study (high dose chemotherapy in breast cancer): No financial compensation. Dr. Reiss reports grants or contracts: Gilead Sciences; ViiV Healthcare; Merck: Investigator-initiated study grants to institution; not related to current work and Participation on a Data Safety Monitoring Board or Advisory Board: Gilead Sciences; ViiV Healthcare; Merck: Honoraria for scientific advisory board participation paid to institution. Dr. Schim van der Loeff has nothing to disclose. Dr. Geerlings has nothing to disclose. This does not alter our adherence to PLOS ONE policies on sharing data and materials.***

5. *One of the noted authors is a group or consortium "HIV Transmission Elimination AMsterdam (H-TEAM) Consortium". In addition to naming the author group, please list the individual authors and affiliations within this group in the acknowledgments section of your manuscript. Please also indicate clearly a lead author for this group along with a contact email address.*

**Reply: H-TEAM consortium members were listed in the acknowledgement section, but we have now added the lead author separately, and added all member affiliations in this section as instructed. Please let us know if this should be formatted differently.**

6. *Please include your full ethics statement in the ‘Methods’ section of your manuscript file. In your statement, please include the full name of the IRB or ethics committee who approved or waived your study, as well as whether or not you obtained informed written or verbal consent. If consent was waived for your study, please include this information in your statement as well.*

**Reply: We now added the ethics statement at the end of the methods section, and updated this statement in the editorial manager system.**

7. *Please review your reference list to ensure that it is complete and correct. If you have cited papers that have been retracted, please include the rationale for doing so in the manuscript text, or remove these references and replace them with relevant current references. Any changes to the reference list should be mentioned in the rebuttal letter that accompanies your revised manuscript. If you need to cite a retracted article, indicate the article’s retracted status in the References list and also include a citation and full reference for the retraction notice.*

**Reply: We** **have now revised and updated the reference list.**

**Editor Comments:**

*Please address the following issues, in addition to responding to the reviewers comments below:*

*A completed COREQ checklist is required, or other relevant checklists listed by the Equator Network, such as the SRQR, to ensure complete reporting*

**Reply: We have now completed and uploaded the COREQ checklist for reporting qualitative research. In addition, as this is a mixed-methods study using both qualitative data as well as observational cohort data, we have also completed and uploaded the STROBE checklist for reporting cohort studies.**

*In the introduction, the second sentence should be changed to read, "…33% were aware…"*

**Reply: We have adapted this sentence as suggested (Introduction, line 32).**

*The statement in the first sentence of the third paragraph needs referencing*

**Reply: We have now added a reference to this sentence (Introduction, line 41).**

*Clarify whether there was any piloting of the questionnaire before use*

**The questionnaire was launched in all participating hospitals at the same time, and was not altered after launching. Before launch, the questionnaire was developed and tested by a senior behavioral scientist, a medical psychologist, an epidemiologist and an infectious disease specialist, based on the Attitude-Social norm-self Efficacy (ASE) model, which is an evidence-based instrument derived from the Theory of Planned Behaviour, to assess behavior and its determinants in healthcare personnel. Further details on the development and dissemination of the questionnaire are explained in the referenced parent study in which it was applied more broadly, of which this manuscript is a part, as well as in the published study protocol which is also referenced (references 20 and 21 in this manuscript). Should the editor wish that we further clarify this process in this manuscript as well, then we are happy to add the aforementioned details in this manuscript.**

*Clarify how haematology guideline authors were identified to invite them to participate in interviews?*

**Reply: We have now further clarified how guideline authors were identified (Methods, Interview design and recruitment paragraph, line 93-94):**

***Authors were identified using author lists of currently published national hematology guidelines.***

In the discussion there are two sentences that need grammatical amendments. My suggestions are to change them to read, “...this is reflected in our observed proportions of people with HIV tested…” and, “…highest among people with types of lymphoma…”. The text should be double checked for any other similar instances.

**Reply: We have now adjusted the sentence following the editor’s suggestions (Discussion, line 352-353):**

***Original:***

***This is reflected in our observed proportions HIV tested, which were highest among types of lymphoma requiring immediate treatment, …***

***Adjusted:***

***This is reflected in our observed proportions of patients tested for HIV, which were highest among patients diagnosed with types of lymphoma requiring immediate treatment, …***

**We have additionally checked the entire manuscript for similar instances, and made similar adjustments in the Introduction section (line 53), Results section (line 154, 156, 321, 322, 324), Discussion section (line 352-353) and Table 3.**

**Reviewers' comments:**
**Reviewer #1:**

Overall, this was a very interesting paper to review. The authors aimed to assess and map factors that influence haematologists HIV testing behaviour, and the results and conclusions align with this.

**Reply: We thank the reviewer for these comments.**

*Areas that would benefit from further detail or discussion:
The 5-point Likert scale that was used - how was scale ranked?*

**Reply: The 5-point Likert scales were ranked with 1 meaning the most negative answer, and 5 meaning the most positive answer. Each question had a specific, most appropriate scale (eg. HIV is common in patients with malignant lymphoma had a scale from ‘definitely not’ to ‘definitely yes’, I tested patients for HIV in the last year had a scale from ‘never’ to ‘very often’ etc.). For each question, the precise scale range is defined in supplementary table 1: Content of online questionnaire on barriers and facilitators for HIV testing among patients with malignant lymphoma. However, we have now additionally clarified this in the main manuscript (Methods, Questionnaire design and recruitment paragraph, line 87):**

***The final questionnaire contained fifteen 5-point Likert scale questions (1 = most negative response, 5 = most positive response), including one on self-reported HIV testing in PWML, two on intention to test, and twelve on attitudes, norms and self-efficacy regarding HIV testing in PWML (S1 Table).***

*Were questionnaire responses anonymous?*

**Reply: Questionnaires were completed anonymously, as had now been clarified (Methods, Questionnaire design and recruitment paragraph, line 82):**

***Online questionnaires on HIV testing in PWML were conducted anonymously among hematologists in June 2020.***

The interviews ranged from 9-30 minutes – the manuscript would benefit from a discussion on this.

**Reply: We thank the reviewer for this interesting suggestion. We hypothesize that the considerable range in duration of interviews may indicate that some hematologists were more interested and involved in the subject and provided more data, whereas others were more succinct. This suggests that the interviewed group was heterogeneous in terms of subject engagement, and further increases the robustness of our findings. We have now commented on this in the manuscript (Discussion, Strengths and limitations paragraph, line 402-406):**

***Including questionnaires and interviews with hematologists provided deeper insight into determinants for HIV testing, in addition to assessing proportions of patients who were tested for HIV. The considerable range in duration of interviews may suggest that the interviewed participants formed a heterogeneous group in terms of subject engagement.***

*It was written that eligible patients were given the opportunity to out of the use of their data - how was this done?*

**Reply: All eligible patients were given the opportunity to opt-out of their data being used through written correspondence. In practice, this meant that all patients received a letter notifying them of this study and its purposes following standard operating procedures, and patients had the opportunity to opt out by returning the response slip or through email. The written correspondence procedure is now clarified in the newly added Ethics statement paragraph (Methods, Ethics statement paragraph, line 132-133).**

*The limited generalisability of the interviews was discussed, however, interviews aren’t necessarily intended to be generalisable.*

**Reply: We thank the reviewer for this observation and we agree. However, we felt that the limited generalizability of the study deserved mention, as not only interviewee’s views on HIV testing were likely specific for this setting; HIV is relatively uncommon in the Netherlands, which likely impacts healthcare provider’s willingness to apply proactive HIV testing strategies. Additionally, as this study was done in a high-income setting with extensive treatment, monitoring and follow-up facilities for patients with malignant lymphoma, the proportions of patients HIV tested stratified by lymphoma type and treatment status may not be generalizable to settings where this is not the case. We have now adjusted this statement to clarify not only the interview responses, but all findings of this study may be of limited generalizability to other settings (Discussion, Strengths and limitations paragraph, line 411-413:**

***Original:***

***Finally, as some of the aspects described by participants are specific to the Dutch setting, generalization of our findings to other settings should only be done with caution.***

***Adjusted:***

***Finally, as some of the findings in this study are specific to the Dutch setting, generalization of our findings to other settings should only be done with caution.***

**Reviewer #2:**

*This is a very nicely designed and presented study that presents the results of an audit of HIV testing in PWML as recommended by guidelines.*

**Reply: We thank the reviewer for these comments.**

*A few minor suggestions for inclusion. In table one add HIV positivity rates.*

**Reply: We thank the reviewer for this comment. However, considering the set-up of this table (i.e. comparing patients tested vs. patients not tested by patient characteristics), as well as the small number of positive patients per category, we feel like adding HIV positivity in this table will not improve its readability. Instead, we suggest that we could change the information on HIV positivity (Results section, HIV testing results paragraph, line 174-177) to a separate table. We had not done so previously, as this manuscript already includes 3 tables and 2 figures. If the Editors feel like this data is indeed better depicted in a table, we will make these changes as suggested.**

Discussion - comparison of responses to survey and those hospitals with lowest test rates. would focus on this - comments on verifying cost-effectiveness not aim of study.

**Reply: We thank the reviewer for this interesting suggestion. In our study, we observed a significant difference in HIV testing rates among patients stratified by hospital of inclusion (range: 49% - 76%). Following the Reviewer’s suggestion, we initially hypothesized this variation may be really due to underlying variation in type of lymphoma by hospital. However, when we stratified by both hospital and type of lymphoma, we found persistent variation among some types, which we have now further elaborated upon (Results section, HIV testing results paragraph, line 156-161):**

***The proportion of patients tested for HIV varied significantly by hospital (Table 1). By type of lymphoma, significant differences in proportion of patients tested by hospital were observed among patients with Hodgkin’s lymphoma (p=0.007), DLBCL (p=0.037), mantle cell lymphoma (p=0.029), follicular lymphoma (p=0.001) and marginal zone or mucosa-associated lymphoid tissue lymphoma (p<0.001), but not among patients with T-cell lymphoma (p=0.364), Burkitt lymphoma (p=0.891), LPL (p=0.504), and other NHL types (p=0.095).***

**When looking at self-reported HIV testing behavior in the past year, we found that lowest scores were found in the non-teaching hospital, while highest scores were observed in the teaching hospitals. We found no difference in self-reported intention to test for HIV in the future by hospital. We have now added these distinctions to our manuscript as well (Results section, Questionnaire results paragraph, line 185-190):**

***By hospital, self-reported testing in the last year varied significantly (p=0.032) and was lowest in the non-teaching hospital (mean Likert score 2.8, SD 1.7), followed by the university hospitals (mean 4.3, SD 0.9), while it was highest in the teaching hospitals (mean 4.4, SD 0.9). Intention to test in the future did not differ significantly (mean Likert scores of 4.6, SD=0.6 in the university hospitals, 4.6, SD=0.9 in the teaching hospitals, and 4.1, SD=4.1* in the non-teaching hospital*, p=0.480).***

**In our patient data, we found the highest HIV testing rate in teaching hospital 1, while the lowest HIV testing rate was observed in teaching hospital 2. However, the HIV testing data was collected from a cohort of patients over 5.5 years, during which time the hematology staff may have changed, while all questionnaires were completed during a one-month period, invalidating a direct comparison of responses to the survey and those hospitals with lowest test rates in our study. As we do not have the data to relate HIV testing from the patient records to self-reported testing from questionnaires by individual hematologist due to the anonymous nature of the questionnaires, we cannot further discuss the comparison of responses to the survey and observed test rates.**

All presented barriers are provider barriers and should be described as such. patient-level barriers are barriers perceived by patients. in this care older age, discomfort etc are provider barriers and should be labelled as such.

**Reply: We thank the reviewer for this important correction. We have now re-labelled the barriers we previously called ‘patient level barriers’ as ‘provider level barriers (related to patients’ characteristics)’ In table 2, in the Results section Interview results paragraph, line 262, 269 and 277, and in the Discussion section line 379.**
